# Independent Effects of Eye and Hand Movements on Visual Working Memory

**DOI:** 10.3389/fnsys.2018.00037

**Published:** 2018-08-17

**Authors:** Nina M. Hanning, Heiner Deubel

**Affiliations:** ^1^Allgemeine und Experimentelle Psychologie, Ludwig-Maximilians-Universität München, Munich, Germany; ^2^Graduate School of Systemic Neurosciences, Ludwig-Maximilians-Universität München, Munich, Germany

**Keywords:** working memory, saccades, reaching movements, motor processes, attention

## Abstract

Both eye and hand movements have been shown to selectively interfere with visual working memory. We investigated working memory in the context of simultaneous eye-hand movements to approach the question whether the eye and the hand movement systems independently interact with visual working memory. Participants memorized several locations and performed eye, hand, or simultaneous eye-hand movements during the maintenance interval. Subsequently, we tested spatial working memory at the eye or the hand motor goal, and at action-irrelevant locations. We found that for single eye and single hand movements, memory at the eye or hand target was significantly improved compared to action-irrelevant locations. Remarkably, when an eye and a hand movement were prepared in parallel, but to distinct locations, memory at both motor targets was enhanced—with no tradeoff between the two separate action goals. This suggests that eye and hand movements independently enhance visual working memory at their goal locations, resulting in an overall working memory performance that is higher than that expected when recruiting only one effector.

## Introduction

Eye and hand movements have been shown to bind visual attention to their goal locations during movement preparation (Kowler et al., 1995; Deubel and Schneider, 1996; Deubel et al., 1998; Rolfs et al., 2013), and it has been suggested that the underlying attentional mechanisms are effector-specific and independent (Jonikaitis and Deubel, 2011; Perry et al., 2016; Hanning et al., 2018), i.e., the attentional benefit at one effector’s movement target is not affected by the concurrent movement preparation of the other effector. Furthermore, both eye (Bays and Husain, 2008; Hanning et al., 2016; Ohl and Rolfs, 2017) and hand movements (Heuer et al., 2017) selectively enhance visual working memory at their action goals, presumably due to the associated deployment of attention (Hanning et al., 2016). Given the assumption of independent mechanisms that drive attention to eye and hand targets, it is conceivable that the two effector systems also separately interact with working memory. We therefore investigated spatial working memory performance in the context of simultaneous eye-hand movements. If eye and hand movements independently of each other enhance working memory at their target locations, any memory benefit observed at the eye target should not be affected by the concurrent preparation of a hand movement, and vice versa.

## Methods

### Participants and Apparatus

Seven right-handed observers (three females, ages 24–32) participated in Experiment 1A, five of whom also completed Experiment 1B (two females, ages 25–32). Seven right-handed observers (three females, ages 24–32) participated in Experiment 2. All participants gave written informed consent. The protocols for the study were approved by and the study was carried out in accordance with the ethical review board of the Faculty of Psychology and Education of the Ludwig-Maximilians-Universität München, in accordance with the Declaration of Helsinki. Gaze position was recorded using an EyeLink 1000 Tower Mount (SR Research, Osgoode, ON, Canada) at a sampling rate of 1 kHz. The experiment was implemented in Matlab (MathWorks, Natick, MA, USA), using the Psychophysics (Brainard, 1997; Pelli, 1997) and EyeLink toolboxes (Cornelissen et al., 2002). Stimuli were presented on a 45° inclined touchscreen (Elo 2700 IntelliTouch, Elo Touchsystems, Menlo Park, CA, USA) with a spatial resolution of 1,280 × 1,024 pixels and a vertical refresh rate of 60 Hz.

### Procedure

#### Experiment 1A: Eye and Hand Movements

**Figure 1:**
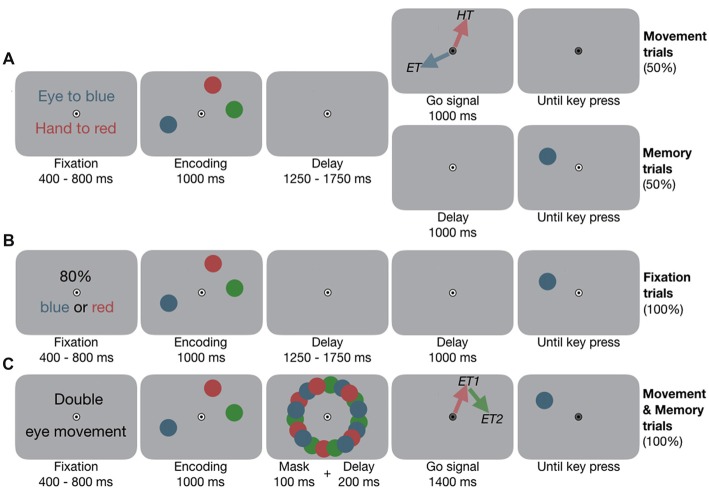
Experimental design. **(A)** Experiment 1A, example condition eye-hand movements (EYE-HAND). Participants fixated the central bull’s eye and placed their index finger slightly below. They encoded the locations of the three colored dots. In *Movement trials*, once the fixation target (FT) changed color, they performed the movements according to pre-block instruction. Red and blue arrows visualize the motor targets (*ET*, eye target; *HT*, hand target) and were not shown in the experiment. In *Memory trials*, the FT did not change color, instead one dot reappeared, and participants performed a location change discrimination task. **(B)** Experiment 1B, example condition two targets (2TAR). At the beginning of each block, participants were informed about which color(s)—either one or two—would be tested in 80% of the cases at the end of the trial. **(C)** Experiment 2, example condition double eye (2EYE). Participants encoded the locations. Once the FT changed color, they performed two successive eye movements towards any two of the memorized locations, e.g., first eye movement (*ET1*) to red, second eye movement (*ET2*) to green. Afterwards, any of the dots reappeared, and participants performed the memory task.

At the beginning of each block, participants were instructed to perform single eye movements (*EYE*), single hand movements (*HAND*) or simultaneous eye-hand movements (*EYE-HAND*) to certain target colors (see Figure 1A). Participants fixated a central fixation target (FT, radius 0.5 deg; deg indicates degrees of visual angle) on gray background, their right index finger remained slightly below the eye fixation. After 400–800 ms, three colored dots (red, green and blue, radius 1 deg) appeared at random angles on an imaginary circle 8 deg around fixation for 1,000 ms. Participants memorized the locations of these dots. After 1,250–1,750 ms, in 50% of the trials (*Movement trials*) the FT turned gray and participants performed the movement(s): for example, in the *EYE* condition, they looked to the location they memorized for the blue dot, in the *HAND* condition, they pointed to the location they memorized for the red dot, and in the *EYE-HAND* condition they looked to the blue and simultaneously pointed to the red location. In this example, the green dot served as a control location that had to be memorized but was not a motor target (colors were counterbalanced across participants). In the other half of trials (*Memory trials*), the FT did not change color and participants kept fixating. Instead, one of the dots reappeared and participants indicated via button press whether its location had changed clockwise or counterclockwise on the imaginary circle. They were instructed to perform the movement(s) as fast and precise as possible, the spatial memory task was not speeded. As *Memory trials* and *Movement trials* were randomly interleaved, participants always prepared the instructed movement(s), even though they actually moved in only half of the trials. This allowed us to investigate the effect of movement preparation on working memory, avoiding potential confounds induced by movement execution. We took location change discrimination performance as a proxy of working memory performance. See Supplementary Information S1 for details about the procedure.

#### Experiment 1B: Fixation Control

To disentangle the influence of attentional load from movement-related effects on working memory in Experiment 1A, we conducted a control experiment in which, instead of performing one or two movements, participants attended to one or two items. At the beginning of each block they were informed which of the memory items would be tested with a higher probability. To resemble the single and combined motor tasks of Experiment 1A, in separate experimental blocks either one (*1TAR*) or two targets (*2TAR*) received an increased likelihood to be tested. Memory task, timing and visual input were equivalent to the *Memory trials* of Experiment 1A (see Figure 1B), but we biased the test likelihood according to the pre-block instruction: in a *1TAR* block, only one item, e.g., the blue one, would re-occur in 80%, while the other two items re-occurred in 20%. In a *2TAR* block the blue or red item would re-occur in 80% of the cases (*2TAR*), while the green item only re-occurred in 20%. See Supplementary Information S1 for details about the procedure.

#### Experiment 2: Double Eye and Double Hand Movements

To assess whether the effects of eye and hand movements are effector-specific and independent of each other, in Experiment 2 we contrasted the effects of two movements—either one of each effector system or two movements within the same system. At the beginning of each block, participants were instructed to perform an eye movement (*EYE*), a hand movement (*HAND*), simultaneous eye-hand movements (*EYE-HAND*), double eye (*2EYE*), or double hand (*2HAND*) movements. After 400–800 ms of fixation, three colored dots (red, green and blue, radius 0.75 deg) appeared at randomly selected angles 8 deg around fixation for 1,000 ms (see Figure 1C). During the first 100 ms of the following 300 ms delay, the items were masked by a circular arrangement of multiple colored dots. Afterwards, the FT turned gray and participants performed the instructed movement(s). In the *2EYE* condition, for example, they could first look to the red, and immediately afterwards to the green location, at free choice. In the *2HAND* condition they instead performed double hand movements. After their movement(s), one of the dots reappeared and participants performed the location change discrimination task. See Supplementary Information S1 for details about the procedure.

### Data Analysis

We detected saccades offline based on the eye velocity distribution (Engbert and Mergenthaler, 2006). In all Experiments we took location change discrimination performance (clockwise or counterclockwise) as a proxy of working memory performance. We initially computed the mean single subject performance for the different locations of each motor condition. For statistical comparisons we conducted permutation tests. We resampled the respective mean individual subject data pairs and derived *p*-values by locating any observed difference on the permutation distribution (difference in means based on 1,000 permutation resamples), next to which we report effect sizes (Cohen’s *d*). To visualize group performance we averaged the individual means across participants.

## Results

### Experiment 1A

**Figure 2:**
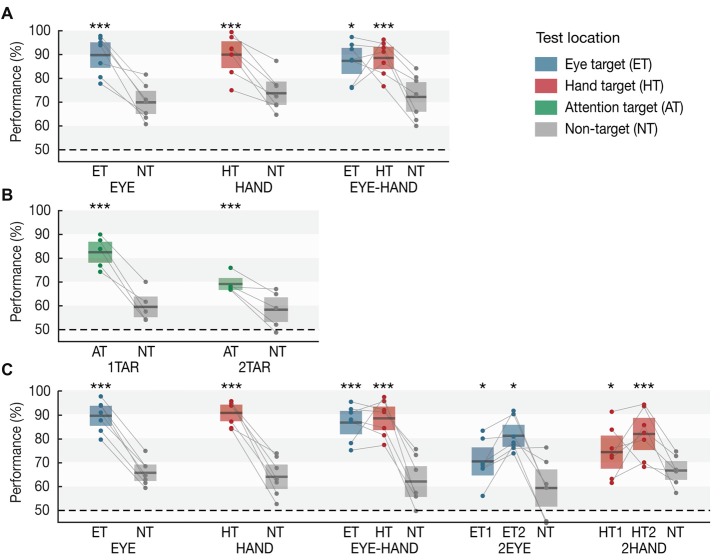
Working memory performance in **(A)** Experiment 1A, **(B)** Experiment 1B and **(C)** Experiment 2 as a function of condition and test location. Horizontal lines within each whisker plot indicate the mean discrimination performance for each condition’s motor targets, attention targets, and non-targets. Colored bars (eye: blue, hand: red, attention target: green, non-target: gray) show 95% confidence intervals. Dots connected by lines represent averaged individual subject data. Asterisks indicate significant differences between targets and the respective condition’s non-targets (*p < 0.05, **p < 0.01, ***p = 0.001).

Results are based on the analysis of the *Memory trials* (see Figure 2A). When participants prepared only an eye movement during the maintenance interval (*EYE*), we observed a clear memory benefit for items memorized at the eye target (*p* = 0.001, Cohen’s *d* = 3.364). Likewise, when only a single hand movement was required (*HAND*), memory at the hand target was superior to motor irrelevant locations (*p* = 0.001, *d* = 2.658). Importantly, when participants simultaneously performed an eye and a hand movement towards different target locations (*EYE-HAND*), we found a memory benefit both at the eye (*p* = 0.039, *d* = 2.240) and the hand target (*p* = 0.001, *d* = 2.578) compared to non-target locations. Importantly, we observed no tradeoff compared to the respective single movement conditions, i.e., performance at each motor target was approximately as high as if just a single eye or hand movement was performed.

### Experiment 1B

In Experiment 1B (Figure 2B), when one item was attended, memory performance for this item was superior to the unattended items (*p* = 0.001, *d* = 3.839). Crucially, when participants payed attention to two items, we observed increased working memory performance for both attended items compared to the one unattended item (*p* = 0.001, *d* = 1.941), however—unlike the *EYE-HAND* condition of Experiment 1A—we also observed a tradeoff: the benefit at two attended items was significantly smaller than the benefit at a single attended item (*p* = 0.001, *d* = 2.723).

### Experiment 2

In Experiment 2 (Figure 2C), we again observed a memory benefit at the motor targets of single *EYE* (*p* = 0.001, *d* = 4.309) or *HAND* movements (*p* = 0.001, *d* = 4.467), as well as at both targets of simultaneous *EYE-HAND* movements (eye target: *p* = 0.001, *d* = 3.159; hand target: *p* = 0.001, *d* = 3.362), again without any tradeoff between the two. When participants performed two eye movements (*2EYE*), we observed a memory benefit at both eye targets compared to the non-target (first target: *p* = 0.042, *d* = 1.254; second target: *p* = 0.013, *d* = 2.623), but memory performance at both was significantly lower compared to the eye target in the *EYE-HAND* condition, i.e., when the eye movement was accompanied by a hand movement instead of a second eye movement. Likewise, in the *2HAND* condition, performance at both hand targets was increased compared to the non-target (first target: *p* = 0.023, *d* = 0.747; second target: *p* = 0.001, *d* = 1.754), but at both targets was significantly lower compared to the hand target in the *EYE-HAND* condition.

## Discussion

When an eye and a hand movement were performed while maintaining spatial information, working memory performance at both motor targets was improved—approximately as much as if just a single eye or hand movement was made. This is surprising, as it is well established that our working memory capacity is limited (e.g., Luck and Vogel, 1997), and current working memory models assume that memory for one item can only be enhanced at the expense of memory for other items stored (e.g., Bays et al., 2009). We observed such typical memory tradeoff when participants attended to one or two items instead of performing movements: the average memory for two attended items was significantly lower than for one attended item—in contrast to what we found for one (eye or hand) compared to two (eye and hand) motor targets, in which case no tradeoff occurred. As the memory benefit at one effector’s movement target was unaffected by the concurrent movement preparation of the other effector, we conclude that eye and hand movements independently of each other enhance working memory.

This finding mirrors the reported independent attentional enhancements at eye and hand targets (Jonikaitis and Deubel, 2011; Hanning et al., 2018), which are thought to result from effector-specific feedback loops between frontoparietal and posterior areas (Perry et al., 2016; Perry and Fallah, 2017). Likewise, visuospatial working memory is assumed to rely on recurrent feedback between prefrontal and posterior cortices (Hale et al., 1996; Chafee and Goldman-Rakic, 2000), and it has been hypothesized that this feedback activity is influenced by motor actions like eye or hand movements (Lawrence et al., 2001). Our data suggest that these movement-evoked effects on working memory are effector-specific: separate feedback signals from the frontoparietal networks serving eye and hand movement preparation may, independently of each other, improve the maintenance of visuospatial information, similar to their effects on visuospatial attention. In consistence with this hypothesis, we found a memory tradeoff between the motor targets of double eye or double hand movements, demonstrating that two movements originating from the same feedback network do not elicit independent memory benefits.

Our results challenge current working memory models that assume an overall limit, be it capacity or resource: contrary to the widespread belief, improved memory for a subpart of the stored content does not necessarily burden memory for the remaining content. Eye and hand movements independently of each other enhance visuospatial memory at their motor targets, resulting in overall memory performance that is higher than that expected when recruiting only one or no effector.

## Author Contributions

NH developed the study concept and wrote the manuscript. HD contributed to the study design and provided critical revisions. Data collection and data analysis were performed by NH. Both authors interpreted the data and approved the final version of the manuscript for submission.

## Conflict of Interest Statement

The authors declare that the research was conducted in the absence of any commercial or financial relationships that could be construed as a potential conflict of interest.
