# Supplementary material for: Independent Effects of Eye and Hand Movements on Visual Working Memory
Source: Front Syst Neurosci. 2018 Aug 17;12:37. doi: 10.3389/fnsys.2018.00037 (PMC6107693; doi:10.3389/fnsys.2018.00037)
Supplement: Supplementary file 1 [file Data_Sheet_1.PDF]

Supplementary Material

Independent effects of eye and hand movements on visual working memory

Nina M. Hanning*, Heiner Deubel

* **Correspondence:** Corresponding Author: hanning.nina@gmail.com

S1. Supplementary Information

Pre-Test

To improve between subject comparability, we conducted a pre-task to adjust the difficulty of the location change task to each participant's memory performance. This pre-task matched the *Memory trials* of the main experiment but participants were fixating throughout the block and we randomly varied the location change angle from trial to trial (7 linearly spaced angles from 0° to 30°, bigger angles correspond to easier discrimination). By fitting psychometric functions to each participant's averaged data, in Experiment 1A and 1B we determined the individual angle corresponding to 80% correct change discrimination performance and used this personalized value for the respective participant during the main task. In Experiment 2, to increase the difficulty of the working memory task in order to avoid ceiling memory performance we used the change angle corresponding to 75% correct discrimination performance.

Trial exclusion criteria

Experiment 1A: We controlled online for broken fixation (eye or finger outside 2.5 degrees of visual angle / deg from the fixation before cue onset), too short (<150 ms) or too long (>650 ms) movement latencies, or movements not landing within 2.5 deg from the respective target.

Experiment 1B: We controlled online for broken eye and finger fixation (outside 2.5 deg from fixation).

Experiment 2: We controlled online for broken eye or finger fixation (outside 2.5 deg from fixation before cue onset), too short (<150 ms) or too long (>1100 ms) movement latencies, or movements not landing within 3.5 deg from the target.

All erroneous trials were repeated in random order at the end of each block.

Block number

Experiment 1A: After training, participants completed 6 blocks of 100 trials in randomized order, two of each condition (*EYE*, *HAND*, and *EYE-HAND*).

Experiment 1B: Participants completed 4 blocks of 75 trials in randomized order, two of each condition (*1TAR* and *2TAR*).

Experiment 2: Participants completed 10 blocks of 70 trials in randomized order, two of each condition (*EYE*, *HAND*, *EYE-HAND*, *2EYE*, and *2HAND*).
